# Personalized Visual Mapping Assistive Technology to Improve Functional Ability in Persons With Dementia: Feasibility Cohort Study

**DOI:** 10.2196/28165

**Published:** 2021-10-19

**Authors:** Jessica Kelleher, Stuart Zola, Xiangqin Cui, Shiyu Chen, Caroline Gerber, Monica Willis Parker, Crystal Davis, Sidney Law, Matthew Golden, Camille P Vaughan

**Affiliations:** 1 Birmingham/Atlanta VA Geriatric Research Education and Clinical Center Atlanta, GA United States; 2 MapHabit, Inc Atlanta, GA United States; 3 Atlanta VA Health Care System Decatur, GA United States; 4 Department of Biostatistics and Bioinformatics Rollins School of Public Health Emory University Atlanta, GA United States; 5 Division of Hospital Medicine Department of Medicine Emory University Atlanta, GA United States; 6 Division of Geriatrics & Gerontology Department of Medicine Emory University Atlanta, GA United States

**Keywords:** aging, ageing, impaired memory, assistive technology, assistive technologies, function, assistive devices, cognition, cognitive, activities of daily living, mobile technology, mobile technologies, dementia, Alzheimer

## Abstract

**Background:**

Mobile health (mHealth) apps using novel visual mapping assistive technology can allow users to develop personalized maps that aid people living with cognitive impairment in the recall of steps needed to independently complete activities of daily living (ADLs), such as bathing, toileting, and dressing.

**Objective:**

This study aims to determine the feasibility and preliminary impact of an mHealth assistive technology app providing guidance to aid individuals living with cognitive impairment in the recall of steps to independently complete ADLs.

**Methods:**

A total of 14 Veterans (mean age 65 SD 9.5 years; 14/14, 100% male; 10/14, 71.4% Black) and 8 non-Veterans (mean age 78, SD 10.3 years; 5/8, 62.5% male; 8/8, 100% Black) were recruited and enrolled from the Department of Veterans Affairs (VA) and non-VA cognitive care clinics. A visual mapping software program, MapHabit, was used to generate a series of personalized visual map templates focused on ADLs created within the MapHabit app. The visual maps were accessed through a tablet device. A 19-item exit questionnaire was administered to the participants to assess perceived improvement in their functional ability after using the MapHabit system for 3 months.

**Results:**

A total of 13 (93%) VA clinic participants and 8 (100%) non-VA clinic participants completed the 3-month study. Baseline cognitive testing indicated impaired to significantly impaired cognitive function. After 3 months of using the MapHabit system, VA clinic participants reported perceived improvement in social engagement (*P*=.01) and performance of ADLs (*P*=.05) compared to the baseline, whereas non-VA clinic participants reported improvements in the performance of ADLs (*P*=.02), mood (*P*=.04), social engagement (*P*=.02), and memory (*P*=.02). All study participants reported they would recommend the MapHabit system to a colleague, and 85% (11/14) of VA and 100% (8/8) of non-VA clinic participants reported a willingness to participate in a future study.

**Conclusions:**

Older VA and non-VA clinic participants with cognitive impairment were willing to use an mHealth app to assist with the completion of ADLs, and they reported positive preliminary effects. A larger study is warranted to assess the efficacy in the setting of a randomized controlled trial.

## Introduction

More than 16 million family members provide unpaid care to a person with Alzheimer disease or other dementias [1]. A recent report estimated that 18.6 billion hours of unpaid care are provided annually, totaling to a value of US $244 billion [1]. In the future, an increase for in-home or institutional care and unpaid assistance by family and friends will be needed as the numbers of those with Alzheimer disease and other forms of cognitive impairment continue to grow. Maintenance of functional ability has been linked to improved quality of life among persons with dementia, and strategies that promote functional ability and independence are of high priority in dementia care research [2,3].

Two recent developments have converged in an attempt to effectively address this challenge. First, developments in the field of assistive technology, including the use of smart devices (eg, tablets, phones, and wearables) have provided ways to enhance the ability of caregivers—both family and professional—to assist individuals with dementia and memory impairment to successfully perform activities of daily living (ADLs), such as bathing, toileting, and dressing [4]. Importantly, there is cumulating evidence that technology adoption is being progressively embraced both among dementia care recipients and their caregivers, findings that bode well for the potential effectiveness of assistive technology interventions [5-7]. Second, developments in the field of neuroscience have shown that there are at least two systems important for memory: the declarative memory system, which is important for conscious recollection of facts and events in our lives, and a more recently recognized procedural, or habit system, that can underlie the development and maintenance of nonconscious habits, motor skills, and other forms of nonconscious procedural learning, often called implicit memory [8]. The present report takes advantage of these two developments in assistive technology and habit memory development and explores the possibility of their use in enhancing the quality of life and functional ability of individuals living with dementia.

MapHabit is a novel mobile health (mHealth) assistive technology app that allows users and/or caregivers to develop personalized maps that aid people living with cognitive impairment in the recall of steps needed to complete the above-described ADLs [4,9]. With repeated use of these maps, people with impaired memory can develop a habit of consulting their visual maps routinely and independently of their caregivers. In this paper, we describe the results from feasibility studies conducted in two clinical samples to determine whether people with cognitive impairment were willing to use the MapHabit system and whether there is evidence of potential benefit to inform a larger, more definitive study.

## Methods

### Study Setting and Ethical Approval

Participants for the feasibility study were recruited from two outpatient clinical programs: a Department of Veterans Affairs (VA) clinic and a non-VA academic health system clinic, focused on serving the needs of older adults with cognitive impairment. The protocol for each clinical cohort was approved by the Emory University Institutional Review Board, and all participants provided written informed consent. The protocols differed primarily based upon the requirement to recruit a care recipient–caregiver dyad in the non-VA clinic population. This provided the opportunity to assess the impact of assistive technology on two separate groups with similar levels of impairment belonging to two different US health systems. Data were analyzed separately for each cohort in order to comply with data information security protocols established by the VA. Here, we present data based on the outcomes assessed among participants living with cognitive impairment in each study group.

### Intervention

**Figure 1:**
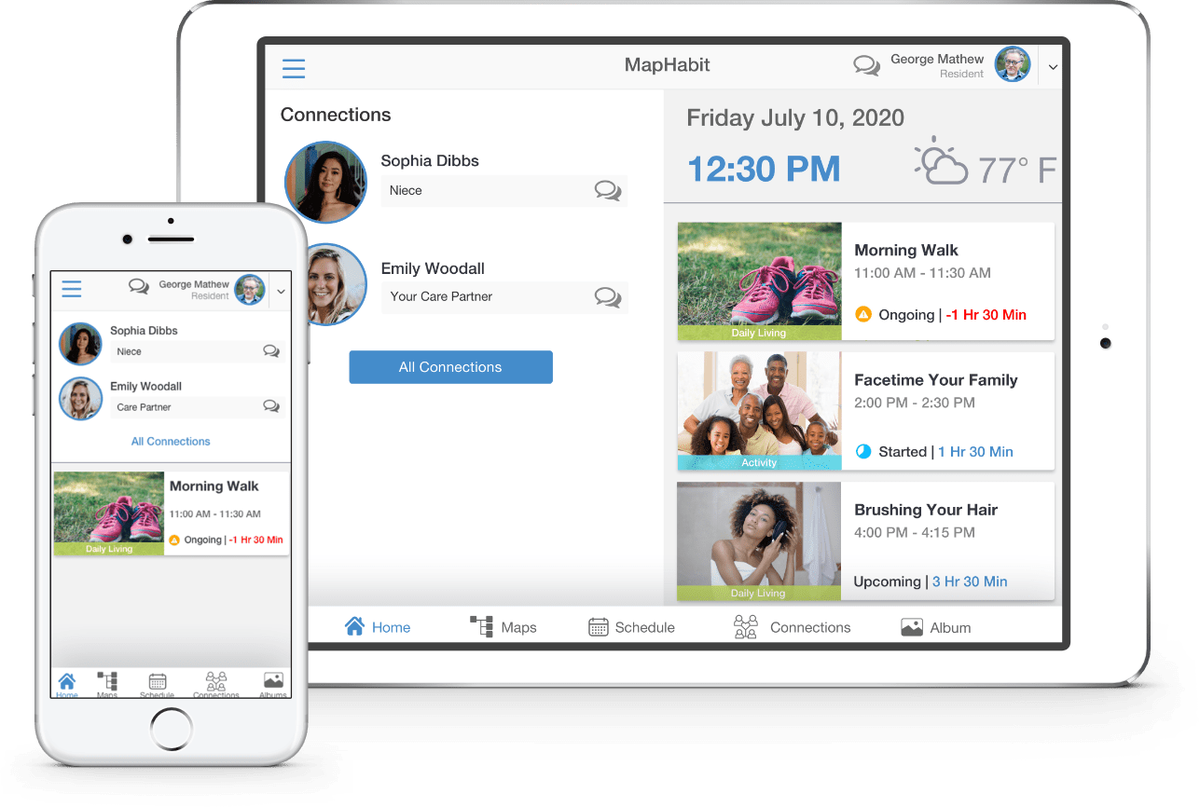
Screenshots of the MapHabit system app on mobile and tablet devices.

A visual mapping software program was used to generate a series of visual map templates created by an assistive technology company, MapHabit, Inc. Participants accessed the software and visual maps through a mobile tablet device. Visual maps consisted of pictures and keywords in a step-by-step sequence to guide and assist participants with memory impairment in organizing and successfully accomplishing ADLs [4]. Participants were encouraged to self-select visual maps based on personal preferences and needs, and they could add images from their own environment to personalize the selected templates. Snapshots of the MapHabit system are presented in Figure 1. Visual maps involving ADL performance were commonly selected, with those pertaining to medication, bathing, and dressing being the most popular. After a staff member conducted initial training and development of selected ADL maps, participants were given an iPad to access and use the MapHabit system.

### Measures

Demographic characteristics included age, sex, self-reported race and ethnicity, and the presence of a caregiver. Baseline cognition was assessed using the Repeatable Battery for the Assessment of Neuropsychological Status (RBANS), a brief, individually administered test measuring attention, language, visuospatial or constructional abilities, and immediate and delayed memory. The RBANS consists of 12 subtests, which yield 5 index scores of the measures described above, and a *total scale score* [10]. A 19-item overall exit questionnaire was administered to assess whether any change (positive or negative) occurred as a result of using the MapHabit system for 3 months. The questionnaire is provided in Multimedia Appendix 1. Questions were grouped to assess the various domains of ADL independence (questions #2, #3, #4, and #5 in the exit questionnaire), mood (questions #1, #7, #8, #9, #10, and #11), social engagement (questions #6, #13, #16, and #17), quality of life (questions #14 and #15), and self-reported memory impairment (question #12). The remaining questions of the measurement tool assessed the participants’ overall satisfaction with the MapHabit system. The questionnaire was administered orally to the participants, using a Likert scale with a self-rating format (5=much better, 4=better, 3=not much change, 2=worse, or 1=much worse). A 2-item yes/no questionnaire assessed participants’ overall user experience with the system.

### Statistical Analysis

Baseline characteristics for each clinical sample were analyzed using descriptive statistics. Exit interview responses were compared using the Wilcoxon signed rank test to assess any differences from an expected result of “not much change”—that is, a score of 3 on the Likert scale for the exit questionnaire. Analyses were conducted using the RStudio statistical package (version 1.1.463).

## Results

In all, 14 VA clinic (mean age 65 SD 9.5 years; 14/14, 100% male; 10/14, 71% Black) and 8 non-VA clinic (mean age 78, SD 10.3 years; 5/8, 62.5% male; 8/8, 100% Black) participants were enrolled in the study (Table 1). Following the 3-month intervention, 13 of the 14 (93%) VA clinic participants and all 8 (100%) non-VA clinic participants completed the study. Baseline cognition measured by the RBANS indicated impaired to significantly impaired cognitive function, on average, across all 5 indices of neuropsychological function [6]. Immediate memory scores indicated impaired ability to remember information immediately after it was presented. Visuospatial or construction scores indicated impaired ability to perceive spatial relations and to construct a spatially accurate copy of a drawing. Language scores indicated impaired ability to respond verbally to either naming or retrieving learned material. Attention scores indicated impaired capacity to remember and manipulate both visually and orally presented information in short-term memory. Delayed memory scores indicated impaired anterograde memory. Total scale scores were calculated by summing the abovementioned 5 index scores.

**Figure 2:**
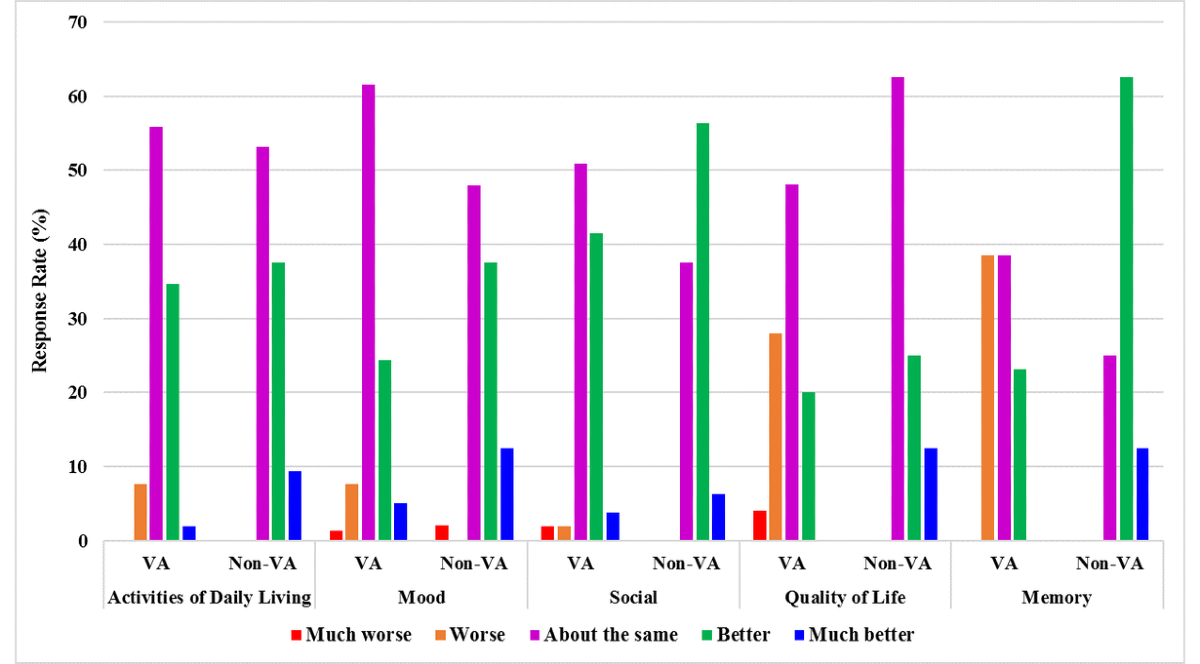
Exit interview responses from study participants 3 months after using personalized visual maps. Non-VA: non–Veterans Affairs clinic participants; VA: Veterans Affairs clinic participants.

After 3 months of using the MapHabit system, analysis of the overall exit questionnaire responses showed that both VA and non-VA clinic participants reported perceived improvement in social engagement (*P*=.01 and *P*=.02, respectively) and performance of ADLs (*P*=.05 and *P*=.02, respectively) compared to the baseline (Figure 2). Non-VA clinic participants also reported perceived improvement in mood (*P*=.04) and memory (*P*=.02; Figure 2). There was no significant perceived change in quality of life for either group (*P*=.09). Many participants reported using a particular map daily, such as to guide teeth-brushing, toileting, or showering. All VA and non-VA clinic participants reported they would recommend the MapHabit system to a colleague, and 85% (11/14) of VA and 100% (8/8) non-VA clinic participants reported a willingness to participate in a future study.

**Table 1:** Baseline characteristics of the Veterans Affairs (VA) and non-VA clinic participants.

Characteristic			VA cognitive clinic (n=14)		Non-VA cognitive clinic (n=8)
Age (years), mean (SD)			65 (9.5)		78 (10.3)
**Gender, n (%)**					
	Female	0 (0)		3 (37.5)	
	Male	14 (100)		5 (62.5)	
**Race, n (%)**					
	White	4 (28.6)		0 (0)	
	Black or African American	10 (71.4)		8 (100)	
	Other	0 (0)		0 (0)	
**Are you of Hispanic or Latino origin, n (%)**					
	Yes	1 (7.1)		0 (0)	
	No	13 (92.9)		8 (100)	
**Marital status, n (%)**					
	Single	3 (21.4)		0 (0.0)	
	Married	9 (64.4)		5 (62.5)	
	Divorced	1 (7.1)		0 (0)	
	Widowed	0 (0)		3 (37.5)	
	Unknown	1 (7.1)		0 (0)	
**Baseline RBANS^a^, mean (SD)**					
	Immediate memory score	70.50 (18.7)		56.75 (20.6)	
	Visuospatial score	67.36 (10.6)		70.12 (19.4)	
	Language score	85.57 (14.2)		71.00 (21.9)	
	Attention score	74.64 (11.9)		69.62 (10.3)	
	Delayed memory score	62.00 (18.6)		52.12 (20.4)	
	Total scale score	63.07 (13.3)		58.00 (15.6)	

^a^RBANS: Repeatable Battery for the Assessment of Neuropsychological Status.

## Discussion

### Principal Findings

The findings from the present studies indicate that the use of visual mapping, specifically the MapHabit system as an assistive technology, is feasible for individuals with memory impairment. Participants reported a positive experience in using the MapHabit system as an assistive technology, adding they would recommend it, and they would again engage with the MapHabit system if the opportunity arose in the future. Furthermore, our study findings suggest that use of the MapHabit system for 3 months may result in perceived improvement in social engagement and performance of ADLs.

Although it has been previously speculated that the use of technology is not widely accepted by older adults, the present results suggest otherwise. Similarly, a study investigating the feasibility of personalized technology use in individuals with mild cognitive impairment found that technology adoption was excellent among both care recipients and their caregivers [5]. Both the present MapHabit system and technology used in the previous study in a senior living community population [5] are personalized, which may suggest that personalization, wherein users are engaged in the selection of activities, serves as a facilitator in technology use among diverse populations of older adults.

Regarding the potential benefit of technology to users’ independence, a recent systematic review of the literature assessing assistive technology use among older adults found that both users and caregivers reported these technologies assisted caregivers by reducing the time, level of assistance, and energy expended on caregiving, as well as anxiety and fear, task difficulty, and safety risk. Previous research has suggested that these improvements were associated with increased independence of the user and reduced need for physical assistance with ADLs [11]. In our studies, both VA and non-VA clinic participants reported perceived improvement in performance of ADLs, echoing the finding that the use of assistive technologies may decrease caregiver burden by promoting increased independence in ADLs by the user.

### Limitations

These studies are not without limitations. As feasibility studies, they were not designed to determine efficacy or definitive evaluation across all outcomes of interest. The outcomes were self-reported; however, patient-reported outcomes are likely the most relevant for determining the initial feasibility of an assistive technology in this population. Although these were not long-term studies, assistive technology appears to be a feasible delivery method for visual maps, and participants were willing to use the technology for at least 3 months. A larger, randomized controlled trial evaluating the visual MapHabit system in individuals living with memory impairment is warranted with a longer follow-up, to determine sustainability.

### Conclusions

Novel findings from this study suggest that the assistive technology MapHabit system is a feasible delivery method for personalized visual maps that can aid people living with cognitive impairment in the recall of steps needed to complete ADLs with greater independence. Strong endorsement from two diverse clinical samples of older adults with cognitive impairment (eg, Black vs White, VA vs non-VA, and male vs female) suggests a potential broad appeal of personalized visual mapping as an assistive technology. Based on research priorities aimed at new strategies to promote ADL independence in individuals with cognitive impairment, the next step entails a definitive study to assess efficacy of personalized visual mapping.

## Appendix

Multimedia Appendix 1 Exit interview questionnaire for caregivers or study participants.

## Abbreviations

ADLs: activities of daily living
mHealth: mobile health
RBANS: Repeatable Battery for the Assessment of Neuropsychological Status.
VA: Department of Veterans Affairs
