# Supplementary material for: Personalized Visual Mapping Assistive Technology to Improve Functional Ability in Persons With Dementia: Feasibility Cohort Study
Source: JMIR Aging. 2021 Oct 19;4(4):e28165. doi: 10.2196/28165 (PMC8564643; doi:10.2196/28165)
Supplement: Multimedia Appendix 1 [file aging_v4i4e28165_app1.docx]

MapHabit Exit Questionnaire Form 1: Caregiver/Participant

Compared to three months ago, before you started using the MapHabit System, please rate each of the following:

1. Mood

5 = Much better. 4 = Better. 3 = About the same. 2 = Worse. 1 = Much worse

1. Independence

5 = Much better. 4 = Better. 3 = About the same. 2 = Worse. 1 = Much worse

1. Ability to carry out some ADLs (eating, dressing, bathing, toileting, etc.)

5 = Much better. 4 = Better. 3 = About the same. 2 = Worse. 1 = Much worse

1. Time it takes to complete ADLs (eating, dressing, bathing, toileting, etc.)

5 = Much better. 4 = Better. 3 = About the same. 2 = Worse. 1 = Much worse

1. Reminders to complete ADLs (eating, dressing, bathing, toileting, etc.)

5 = Much better. 4 = Better. 3 = About the same. 2 = Worse. 1 = Much worse

1. Social interaction

5 = Much better. 4 = Better. 3 = About the same. 2 = Worse. 1 = Much worse

1. Depression

5 = Much better. 4 = Better. 3 = About the same. 2 = Worse. 1 = Much worse

1. Anxiety

5 = Much better. 4 = Better. 3 = About the same. 2 = Worse. 1 = Much worse

1. Frustration

5 = Much better. 4 = Better. 3 = About the same. 2 = Worse. 1 = Much worse

1. Anger

5 = Much better. 4 = Better. 3 = About the same. 2 = Worse. 1 = Much worse

1. Coping ability

5 = Much better. 4 = Better. 3 = About the same. 2 = Worse. 1 = Much worse

1. Memory

5 = Much better. 4 = Better. 3 = About the same. 2 = Worse. 1 = Much worse

1. Social engagement

5 = Much better. 4 = Better. 3 = About the same. 2 = Worse. 1 = Much worse

1. Quality of life

5 = Much better. 4 = Better. 3 = About the same. 2 = Worse. 1 = Much worse

1. Enjoyment of life

5 = Much better. 4 = Better. 3 = About the same. 2 = Worse. 1 = Much worse

1. Expressions of appreciation

5 = Much better. 4 = Better. 3 = About the same. 2 = Worse. 1 = Much worse

1. Cooperation

5 = Much better. 4 = Better. 3 = About the same. 2 = Worse. 1 = Much worse

1. How satisfied are you with your progress in this program?

3 = Completely. 2 = Somewhat. 1 = Not at all

1. Compared to three months ago, before you started the program, do you feel you are:

5 = Much better. 4 = Better. 3 = About the same. 2 = Worse. 1 = Much worse

1. Would you recommend MapHabit to your friends/colleagues? 2 = Yes. 1 = No
2. If there were a follow-up study using MapHabit, would you participate? 2 = Yes. 1 = No
3. What do you think might make MapHabit better?
